# Editorial: Current approaches in infectious disease drug discovery

**DOI:** 10.3389/fchem.2022.1102402

**Published:** 2022-11-30

**Authors:** Floriano P. Silva-Jr, Siva S. Panda, Carolina Horta Andrade, Nicholas Furnham

**Affiliations:** ^1^ LaBECFar - Laboratório de Bioquímica Experimental e Computacional de Fármacos, Instituto Oswaldo Cruz, Fundação Oswaldo Cruz, Rio de Janeiro, Brazil; ^2^ Department of Chemistry and Physics, Augusta University, Augusta, GA, United States; ^3^ LabMol - Laboratory for Molecular Modeling and Drug Design, Faculty of Pharmacy, Universidade Federal de Goiás, Goiania, Brazil; ^4^ Department of Infection Biology, London School of Hygiene and Tropical Medicine, London, United Kingdom

**Keywords:** drug discovery, infectious diseases, neglected tropical diseases, emerging infectious diseases, drug treatments

Goal 3 of the United Nations Sustainable Development Goals is ensuring healthy lives and promoting wellbeing at all ages (WHO, 2015). A major target proposed under this goal is to end the epidemics of AIDS, *tuberculosis*, malaria, and neglected tropical diseases and to combat hepatitis, water-borne diseases and other communicable diseases by 2030. Key to achieving this target is the rapid development of new, safer, and more efficacious treatments against infectious diseases.

The year 2020 has taught us that although well-known infectious disease such as AIDS, malaria, and *tuberculosis* are still present and affecting millions of people worldwide, new and re-emerging infectious diseases can also pose a serious global threat to countries’ health systems and economies. In fact, the full impact of interrupting normal healthcare and health-related research activities combined with the social and economic effects of the COVID-19 pandemic is yet to be seen, but some authors anticipate an increase in reinfections and delay in treatment, which will drag on for years to come (Chaumont et al., 2020).

Despite the rise in the profile and importance of having therapeutic interventions to treat infectious diseases after the past 3 years of the COVID-19 pandemic, we are still struggling with the latter and it is still a challenge to gain sufficient capacity in developing new therapeutics for endemic infectious diseases. There are significant challenges remaining to translate findings and progress innovations from academia to industry and the clinic. This is particularly true of the neglected tropical diseases highlighted in this Research Topic, such as Chagas Disease, fungal infections, viral infections, malaria and resistant bacteria. It is vital that the lessons learned from the COVID-19 pandemic are not lost and are capitalized upon to further rapid development of new interventions for these disease areas using the academic/industry collaborations and new ways of working generated in the pandemic response.

Drug discovery and development is a long and highly expensive process, usually taking 10–15 years from the early discovery of a molecular target or disease-relevant pathway to the final approval of a candidate small molecule to be used in the clinic. Infectious diseases pose many additional challenges within this pathway in addition to the frequent emergence of drug resistance. Several pathogens are associated with the alarming increase in rates of drug resistance and therapeutic failure. Infectious diseases remain devastating in poor and undeveloped countries, with nearly one million deaths yearly. Many infectious are in urgent need of effective and selective therapeutic agents due to multi-drug resistance microorganisms with limited therapeutic options. The development of novel anti-infective drugs with high potency and minimal resistance is the fundamental point of research today.

The goal of this Research Topic was to gather contributions reporting the most up-to-date approaches aimed at the research and development of new drug treatments against established, new, and re-emerging infectious diseases. We aimed to provide the reader a wide view of modern and inventive strategies to accelerate the discovery of new drugs to tackle the infectious diseases burden, moving towards fulfilling the UN’s Sustainable Development Goals by 2030.

In this Research Topic we had five important contributions to the field comprising both review articles and original research papers. Contributions came from authors based on Latin America, Asia and Europe, highlighting the global reach of the theme. Starting with the original research articles, Wang et al. report on the design, and synthesis of novel 1,2,4-triazole-thioether compounds, *in vitro* antifungal activity against eight plant pathogens, as potential inhibitors of cytochrome bc1 complex, an important target enzyme to inhibit the growth of plant pathogens. Moreover, in order to design new and more promising compounds, the authors have developed three-dimensional quantitative structure-activity relationship models (3D-QSAR), using the comparative molecular field analysis (CoMFA) method. In addition, molecular docking revealed that there were hydrophobic interactions between the target compounds and the key favorable residues of cytochrome bc1 complex.

The research article by Ogawa et al. reports on the search of new natural products with antiviral activity using a classification model. Triterpenes are natural products that exhibit anti-herpes simplex virus type 1 (HSV-1) activity. To explore and expand the information about those compounds against HSV-1, authors constructed a predictive classification model for the anti-HSV-1 activity of triterpenes using machine learning methods. As a result, 20 triterpenes were selected and examined for their anti-HSV-1 activity by using a plaque reduction assay, and four compounds exhibited significant *in vitro* activity against HSV-1.

Among reviews articles, Oliveira’s et al. summarize the efforts over the past ∼10 years in finding and developing new chemical entities to treat Chagas disease caused by the protozoan parasite *Trypanosoma cruzi*. They focus their review on the research (mostly from academia) generated by researchers in Latin America, the principal endemic region. Despite the many positive contributions, they identified several limitations to fully capitalize on the major challenge of developing new chemical entities. These include regional expertise gaps in ADME assays and integrated PK/disease model studies as well as the availability of multiparametric data from a structured screening cascade or guided project decisions based on a clear target product profile. The authors appealed for improved data-sharing and organization of multidisciplinary collaborations through implementing regional ADME platforms and by integrating DMPK into early drug discovery projects to increase the chances of identifying novel clinical candidates for the treatment of Chagas disease.

The other mini-review on this topic by Yan et al. focuses on the role of Lipoic acid (LA) in cellular metabolism in *Plasmodium falciparum* and *Staphylococcus aureus*. The mini-review focused on the importance of LA metabolism in posttranslational modification (PTM) and how it can be a chemotherapeutic target for various diseases (*Front. Chem.* 9:742,175). Finally, Santos et al. discuss in deep hydroxymethylation reactions as a straightforward approach to enhance pharmacodynamic and pharmacokinetic properties of lead compounds with emphasis on the group’s own experience with a compound targeting Chagas disease.

In conclusion, we´d like to thank all authors that contributed to this Research Topic and invite the reader to explore the excellent articles in this compilation. We need to be prepared for the next epidemic and pandemic and for this, we need continuous funding and awareness for infectious diseases, specially the most neglected and emerging ones.
